# Supplementary material for: Comparative genomics provides new insights into the diversity, physiology, and sexuality of the only industrially exploited tremellomycete: Phaffia rhodozyma
Source: BMC Genomics. 2016 Nov 9;17:901. doi: 10.1186/s12864-016-3244-7 (PMC5103461; doi:10.1186/s12864-016-3244-7)
Supplement: Additional file 6: — List of orphan genes with links to PFAM (related to Additional file 1: Table S1). (ZIP 1428 kb) [file 12864_2016_3244_MOESM6_ESM.zip › BLAST_HTML_FTR/G04771_P.html]

BLAST Search Results


```
BLASTP 2.2.27+


Reference:
Stephen F. Altschul, Thomas L. Madden, Alejandro A. Schäffer,
Jinghui Zhang, Zheng Zhang, Webb Miller, and David J. Lipman (1997),
"Gapped BLAST and PSI-BLAST: a new generation of protein database
search programs", Nucleic Acids Res. 25:3389-3402.


Reference for
composition-based statistics:
Alejandro A. Schäffer, L. Aravind, Thomas L. Madden, Sergei
Shavirin, John L. Spouge, Yuri I. Wolf, Eugene V. Koonin, and
Stephen F. Altschul (2001), "Improving the accuracy of PSI-BLAST
protein database searches with composition-based statistics and
other refinements", Nucleic Acids Res. 29:2994-3005.


Database: nr
           71,551,133 sequences; 26,053,659,533 total letters


Query= G04771_P

Length=93
                                                                      Score     E
Sequences producing significant alignments:                          (Bits)  Value

emb|CDZ98421.1|  Cyclin PHO80-like [Xanthophyllomyces dendrorhous]     196    9e-61
emb|CDZ98407.1|  Cyclin PHO80-like [Xanthophyllomyces dendrorhous]    87.4    2e-18
emb|CED85056.1|  Cyclin PHO80-like [Xanthophyllomyces dendrorhous]    46.2    6e-04
emb|CED84985.1|  hypothetical protein [Xanthophyllomyces dendrorh...  41.6    0.067
emb|CDJ49244.1|  hypothetical protein, conserved [Eimeria brunetti]   35.4    4.5  


 >emb|CDZ98421.1| Cyclin PHO80-like [Xanthophyllomyces dendrorhous]
Length=239

 Score =  196 bits (499),  Expect = 9e-61, Method: Compositional matrix adjust.
 Identities = 92/92 (100%), Positives = 92/92 (100%), Gaps = 0/92 (0%)

Query  1    MVSDVCYANAAFIRHTPWTLEEINSMEAELLVHIQYRSFISPVCLLRRAKDFWAQPHEDT  60
            MVSDVCYANAAFIRHTPWTLEEINSMEAELLVHIQYRSFISPVCLLRRAKDFWAQPHEDT
Sbjct  148  MVSDVCYANAAFIRHTPWTLEEINSMEAELLVHIQYRSFISPVCLLRRAKDFWAQPHEDT  207

Query  61   HSPGDEDDDDDDDDNDETPFGPTLSSYDILSG  92
            HSPGDEDDDDDDDDNDETPFGPTLSSYDILSG
Sbjct  208  HSPGDEDDDDDDDDNDETPFGPTLSSYDILSG  239


>emb|CDZ98407.1| Cyclin PHO80-like [Xanthophyllomyces dendrorhous]
Length=305

 Score = 87.4 bits (215),  Expect = 2e-18, Method: Compositional matrix adjust.
 Identities = 42/73 (58%), Positives = 51/73 (70%), Gaps = 2/73 (3%)

Query  1    MVSDVCYANAAFIRHTPWTLEEINSMEAELLVHIQYRSFISPVCLLRRAKDFWAQPHEDT  60
            M+SD CYAN AF+R TPW+LEEINSMEAE+L HIQY+S+I P  LL  AK FWA  H++T
Sbjct  232  MISDACYANIAFVRRTPWSLEEINSMEAEMLGHIQYKSYIPPCFLLCLAKAFWA--HQET  289

Query  61   HSPGDEDDDDDDD  73
                   D+   D
Sbjct  290  GIVRARRDNTSSD  302


>emb|CED85056.1| Cyclin PHO80-like [Xanthophyllomyces dendrorhous]
Length=233

 Score = 46.2 bits (108),  Expect = 6e-04, Method: Compositional matrix adjust.
 Identities = 22/52 (42%), Positives = 31/52 (60%), Gaps = 0/52 (0%)

Query  1    MVSDVCYANAAFIRHTPWTLEEINSMEAELLVHIQYRSFISPVCLLRRAKDF  52
            M +D  Y N++F+  T WTL +IN ME ELL  + +  ++ P   L RAK F
Sbjct  177  MSTDNSYHNSSFVDRTSWTLNQINRMETELLGWLSFELYLEPWEFLERAKAF  228


>emb|CED84985.1| hypothetical protein [Xanthophyllomyces dendrorhous]
Length=1087

 Score = 41.6 bits (96),  Expect = 0.067, Method: Compositional matrix adjust.
 Identities = 18/40 (45%), Positives = 24/40 (60%), Gaps = 0/40 (0%)

Query  2    VSDVCYANAAFIRHTPWTLEEINSMEAELLVHIQYRSFIS  41
            VS+  + N AF+  TPWT  EINS+E EL   + Y  + S
Sbjct  191  VSEDSFHNTAFVSRTPWTCGEINSIEKELFARLDYNLYFS  230


>emb|CDJ49244.1| hypothetical protein, conserved [Eimeria brunetti]
Length=667

 Score = 35.4 bits (80),  Expect = 4.5, Method: Composition-based stats.
 Identities = 15/33 (45%), Positives = 21/33 (64%), Gaps = 2/33 (6%)

Query  58  EDTHSPGDEDDDDDDDDNDET--PFGPTLSSYD  88
           EDT++PG+E  D D D+ DE   P  PT+  +D
Sbjct  64  EDTNNPGEESSDTDGDETDEELPPLSPTVEKFD  96


Lambda      K        H        a         alpha
   0.323    0.139    0.450    0.792     4.96 

Gapped
Lambda      K        H        a         alpha    sigma
   0.267   0.0410    0.140     1.90     42.6     43.6 

Effective search space used: 646378144620


  Database: nr
    Posted date:  Sep 23, 2015 12:05 AM
  Number of letters in database: 26,053,659,533
  Number of sequences in database:  71,551,133


Matrix: BLOSUM62
Gap Penalties: Existence: 11, Extension: 1
Neighboring words threshold: 11
Window for multiple hits: 40
```
